# Supplementary material for: High antibody levels and reduced cellular response in children up to one year after SARS-CoV-2 infection
Source: Nat Commun. 2022 Nov 28;13:7315. doi: 10.1038/s41467-022-35055-1 (PMC9701757; doi:10.1038/s41467-022-35055-1)
Supplement: Supplementary file 1 — Supplementary Information [file 41467_2022_35055_MOESM1_ESM.pdf]

Supplementary Figure 1

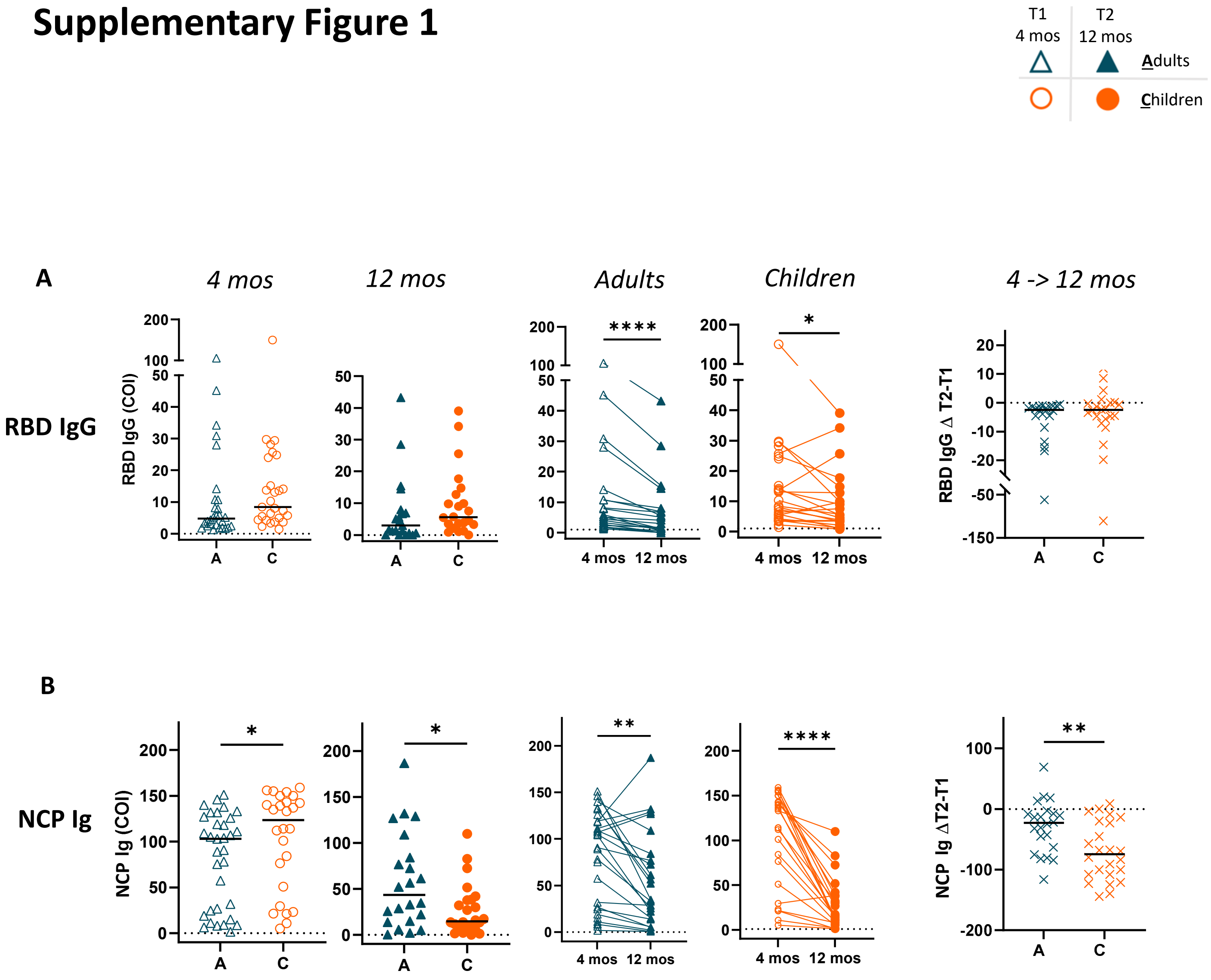

Supplementary Figure 1. Similar decrease of specific antibody levels to receptor-binding domain of spike protein (RBD) in children and adults; faster loss of antibodies to nucleocapsid protein (NCP) in children. Only seropositive subjects at T1 are plotted (31 adults, 27 children; for definition of seropositivity see Methods). Antibodies reactive to SARS-CoV-2 RBD IgG (A) and NCP Ig (B) were measured with Siemens Healthineers and Roche Elecsys, respectively at T1 (4 months after diagnosis; empty symbols) and T2 (12 months after diagnosis; filled symbols) in adults (A; blue symbols) and children (C; orange symbols); dotted line represents a cut-off value for reactivity of cut-off index (COI=1). In the far right graphs a difference between values at T1 and T2 is shown (T1 values subtracted from T2 ones); the dotted line depicts a null difference. Mann-Whitney test and Wilcoxon matched-pairs signed rank test were used for comparing median values (black lines) between adults and children, and between T1 and T2, respectively. Statistical significance was defined as * p≤0.05, **p≤0.01, ***p≤0.001; only statistically significant differences are marked. Source data are provided as a Source Data file.

Supplementary Figure 2

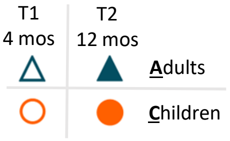

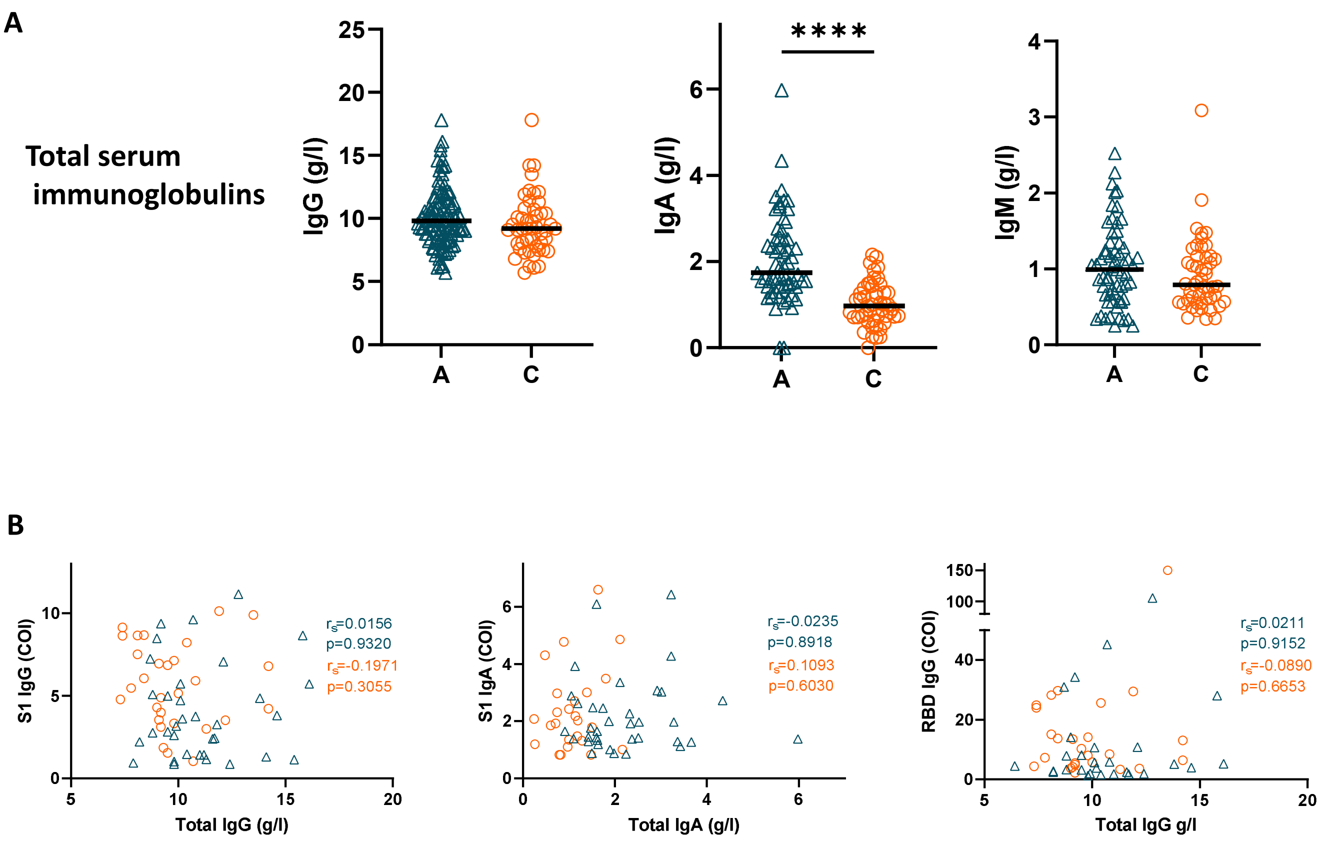

Supplementary Figure 2. Relation between specific antibody levels and total immunoglobulin levels. (A) Total serum antibody levels (IgG, IgA, IgM) in all participants are shown. (B) There is no correlation between the specific SARS-CoV-2 antibodies and total antibody levels in seropositive participants (31 adults,27 children). Mann-Whitney test was used for comparing median values (black lines) between adults and children, statistical significance was defined as ****p≤0.0001; only statistically significant differences are marked. Non-parametric Spearman correlation was applied. Source data are provided as a Source Data file.

Supplementary Figure 3

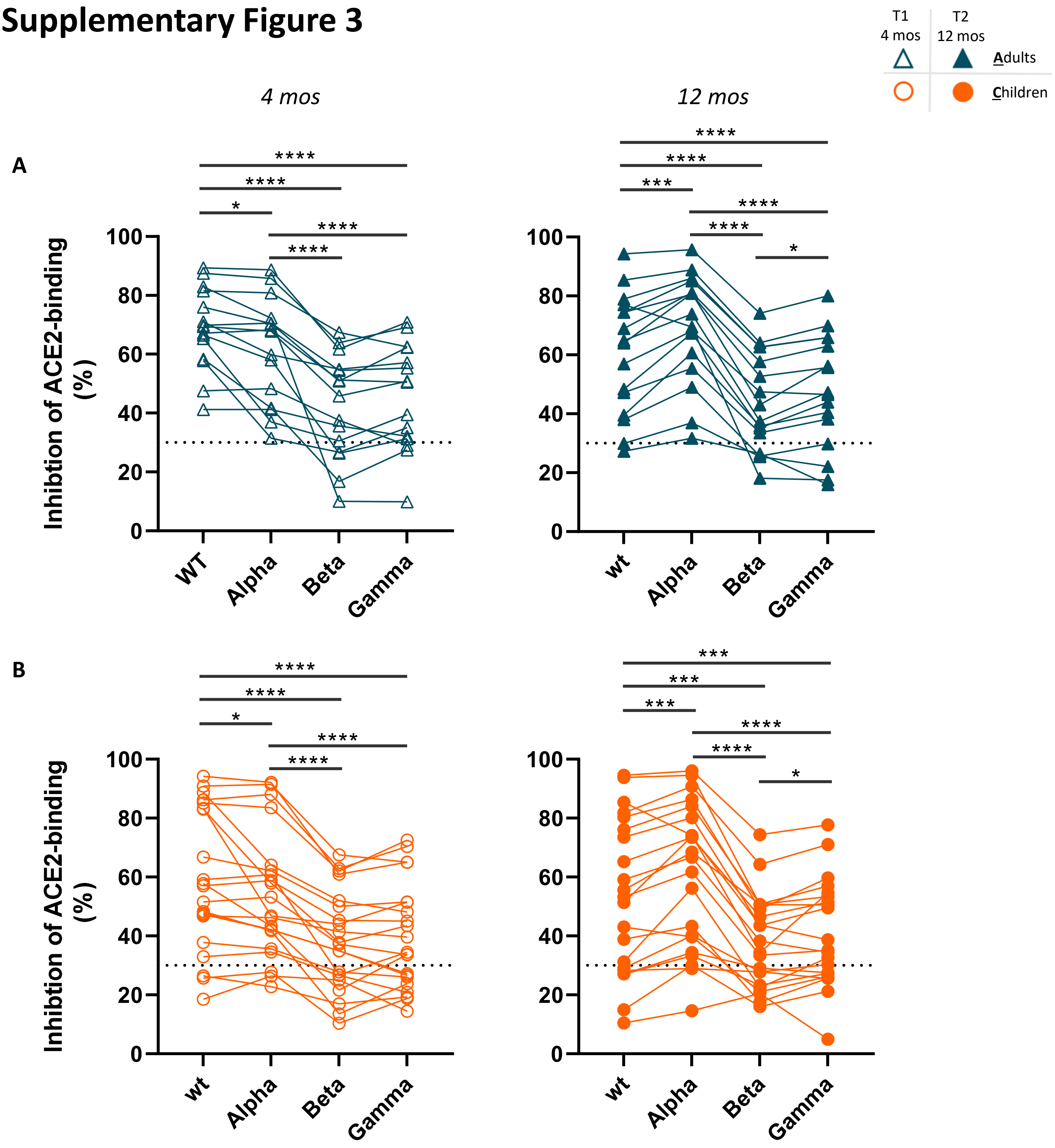

Supplementary Figure 3. Longitudinal decrease in neutralizing capacity against the original Wuhan (WT) strain. Only participants who were seropositive at T1 (for definition of seropositivity see Methods) and who were tested in T2 (excluding vaccinated individuals) are plotted (16 adults, 21 children). Neutralization serum capacity against parental (WT, Wuhan) and Variants of Concern (VOC): Alpha (B.1.1.7), Beta (B.1.351) and Gamma (P.1) strains was tested with the AcroNT SARS-CoV-2 Surrogate Virus Neutralization Test (sVNT*) at T1 (4 months after diagnosis; empty symbols) and T2 (12 months after diagnosis; filled symbols) in adults (A; blue symbols) and children (B; orange symbols); dotted line represents a cut-off value for neutralization (30%). The Wilcoxon matched-pairs signed rank test was used for comparing median values between VOCs. Statistical significance was defined as * p≤0.05, **p≤0.01, ***p≤0.001; only statistically significant differences are marked. Source data are provided as a Source Data file.

Supplementary Figure 4

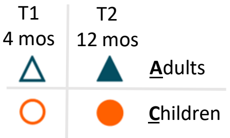

A **Gating strategy (extended B-cell phenotype)**

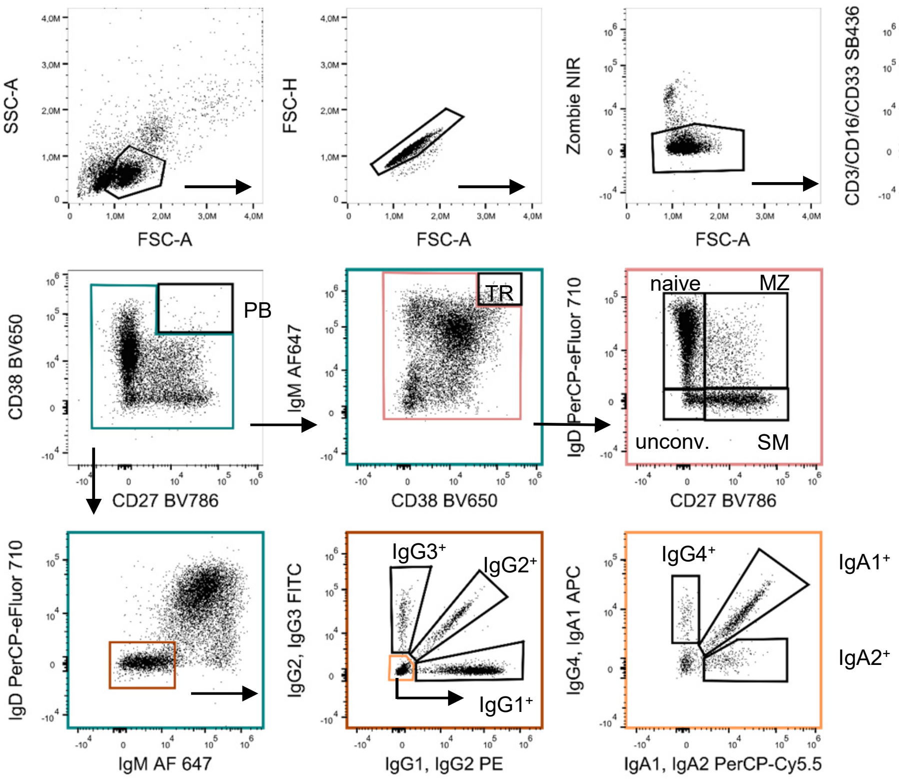

B *All (4 mos)*

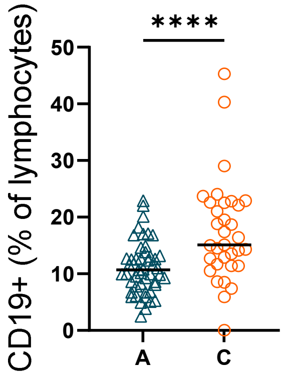

Supplementary Figure 4. Extended B-cell phenotype in adults and children. (A) Gating strategy for B-cell phenotyping of seropositive participants with flow cytometry (for definition of seropositive and details of flow cytometry measurement see Methods). (B) B-cell count for T1 (4 months) is depicted (49 adults with blue triangles, 33 children with orange circles) Abbreviation: TR: transitional B cells, PB: plasmablasts; SM: switched memory B cells; Unconv.: unconventional memory B cells; MZ: marginal zone B cells; IgG1-4: subclasses of IgG; IgA1-2: subclasses of IgA. Mann-Whitney test was used for comparing median values (black lines), statistical significance was defined as ****p≤0.0001. Source data are provided as a Source Data file.

Supplementary Figure 5

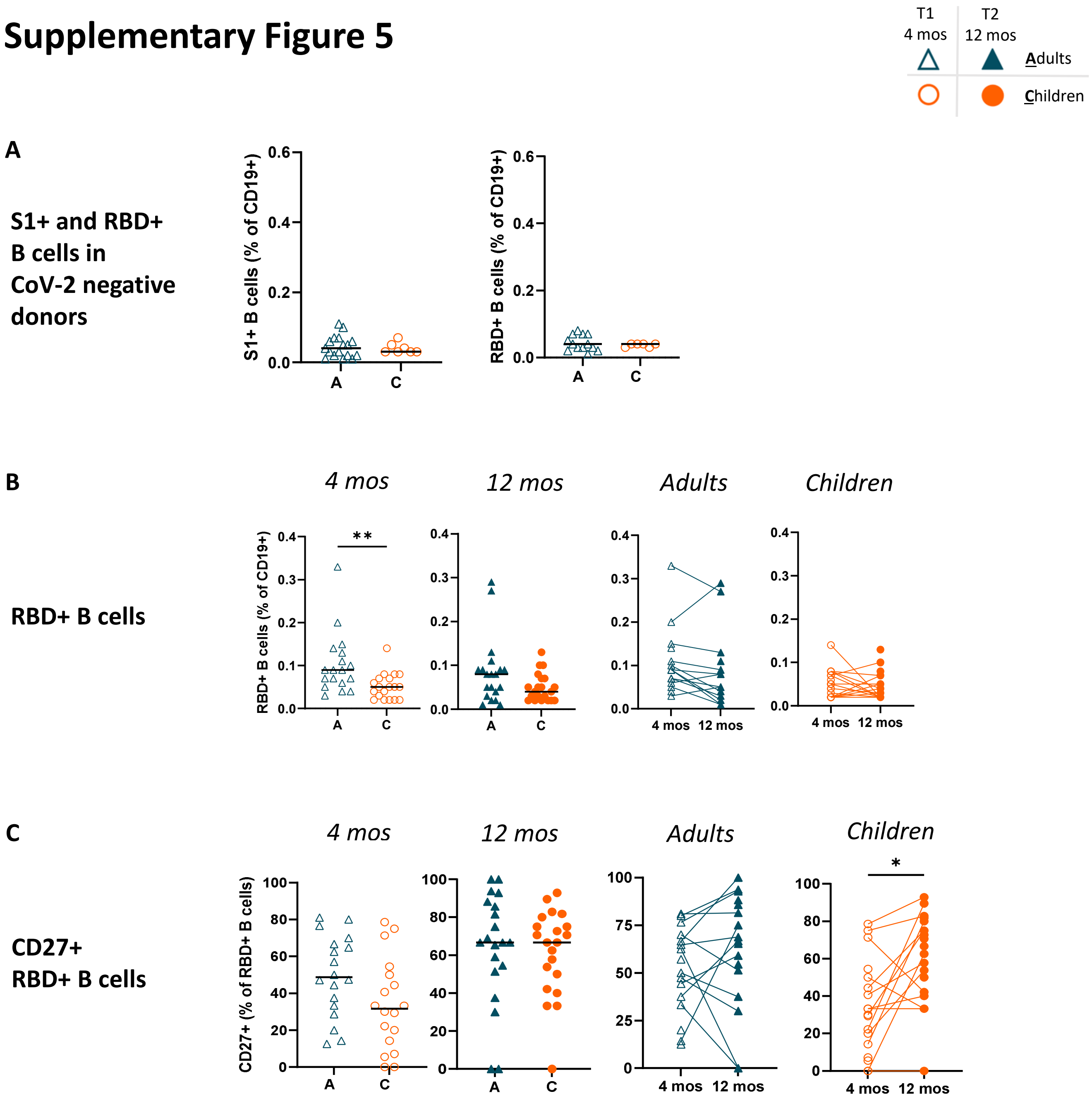

Supplementary Figure 5. Stable low amount of circulating RBD+ B cells with increasing proportion of B cells with memory phenotype in children (similary to S1+ B cells). (A) S1+ and RBD+ B cells were measured in SARS-CoV-2 negative individuals (for definition of seronegativity see Methods) with flow cytometry at T1 (4 months after diagnosis; empty symbols) in adults (A; blue symbols) and children (C; orange symbols). (B) RBD+ B cells for both age groups in SARS-CoV-2 seropositive individuals (for defition of seropositivity see Methods) at T1 and T2 (12 months after diagnosis, filled symbols) are shown. (C) Proportion of CD27+ RBD+ B cells measured with flow cytometry at T1 and T2 in SARS-CoV-2 seropositive individuals. Mann-Whitney test and Wilcoxon matched-pairs signed rank test were used for comparing median values (black lines) between adults and children, and between T1 and T2, respectively. Statistical significance was defined as * p≤0.05, **p≤0.01; only statistically significant differences are marked. Source data are provided as a Source Data file.

Supplementary Figure 6

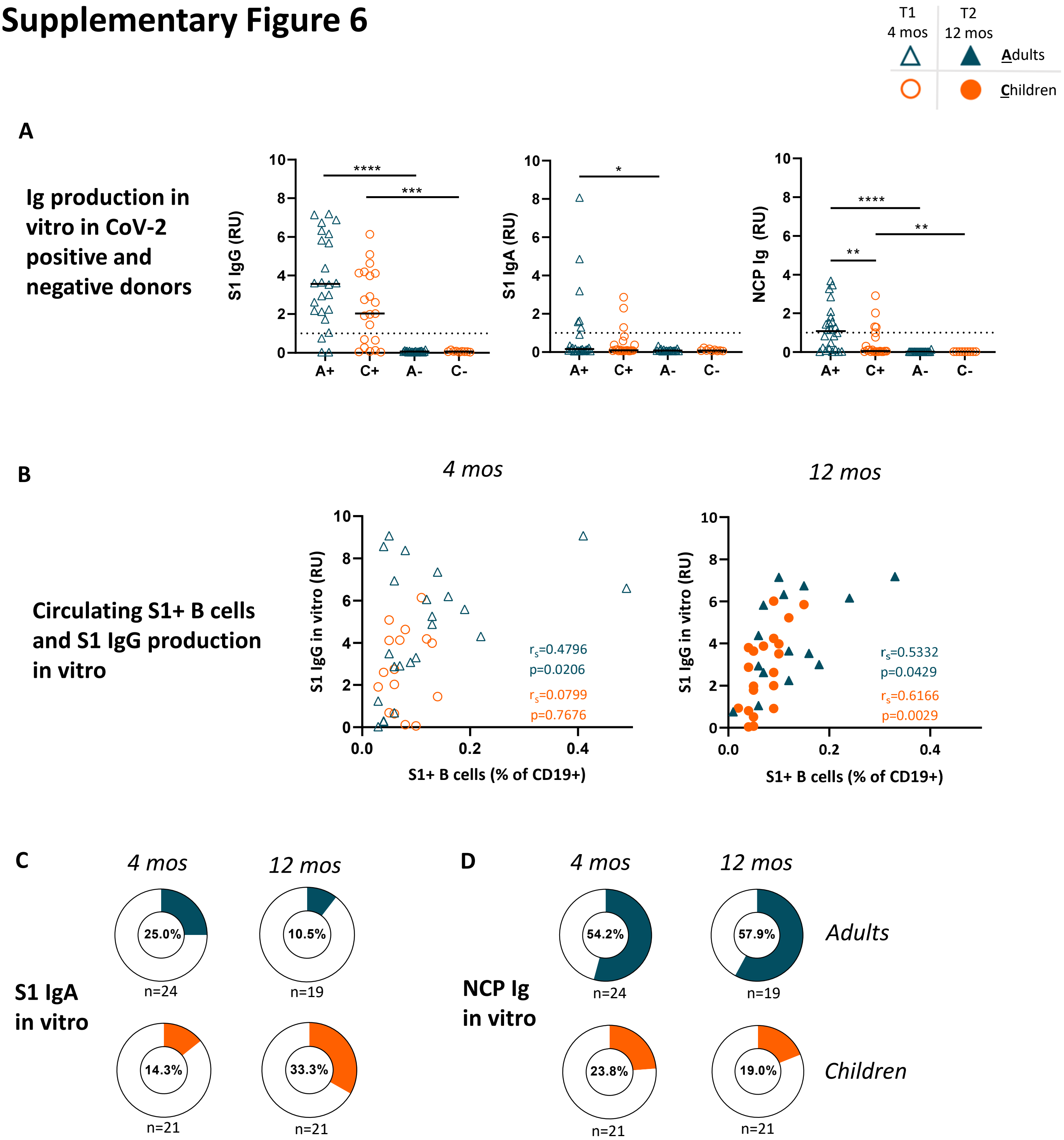

Supplementary Figure 6. Detection of specific antibodies from circulating memory B cells after TLR9 stimulation in vitro. (A) Comparison of antibody production in vitro between SARS-CoV-2 seropositive adults /A+/ and children /C+/ and seronegative ones /A-, C-/ in T1 (for definition of seronegativity see Methods), the dotted line represents a cut-off value for reactivity. Mann-Whitney test was used for comparing median values (black lines) between adults and children, and between T1 and T2, respectively. Statistical significance was defined as * p≤0.05, **p≤0.01; only statistically significant differences are marked. (B) Correlation between circulating S1+ B cells and specific immunoglobulin production in vitro. Non-parametric Spearman correlation was applied. Proportion of participants with proven production of S1 IgA (C) and NCP Ig (D) after stimulation of mononuclear cells (MNC) with TLR9 agonist in vitro at T1 and T2 in subjects who were SARS-CoV-2 seropositive in T1 (for definition of seropositivity and methodic details see Methods) were measured (adults in blue, children in orange). Source data are provided as a Source Data file.

Supplementary Figure 7

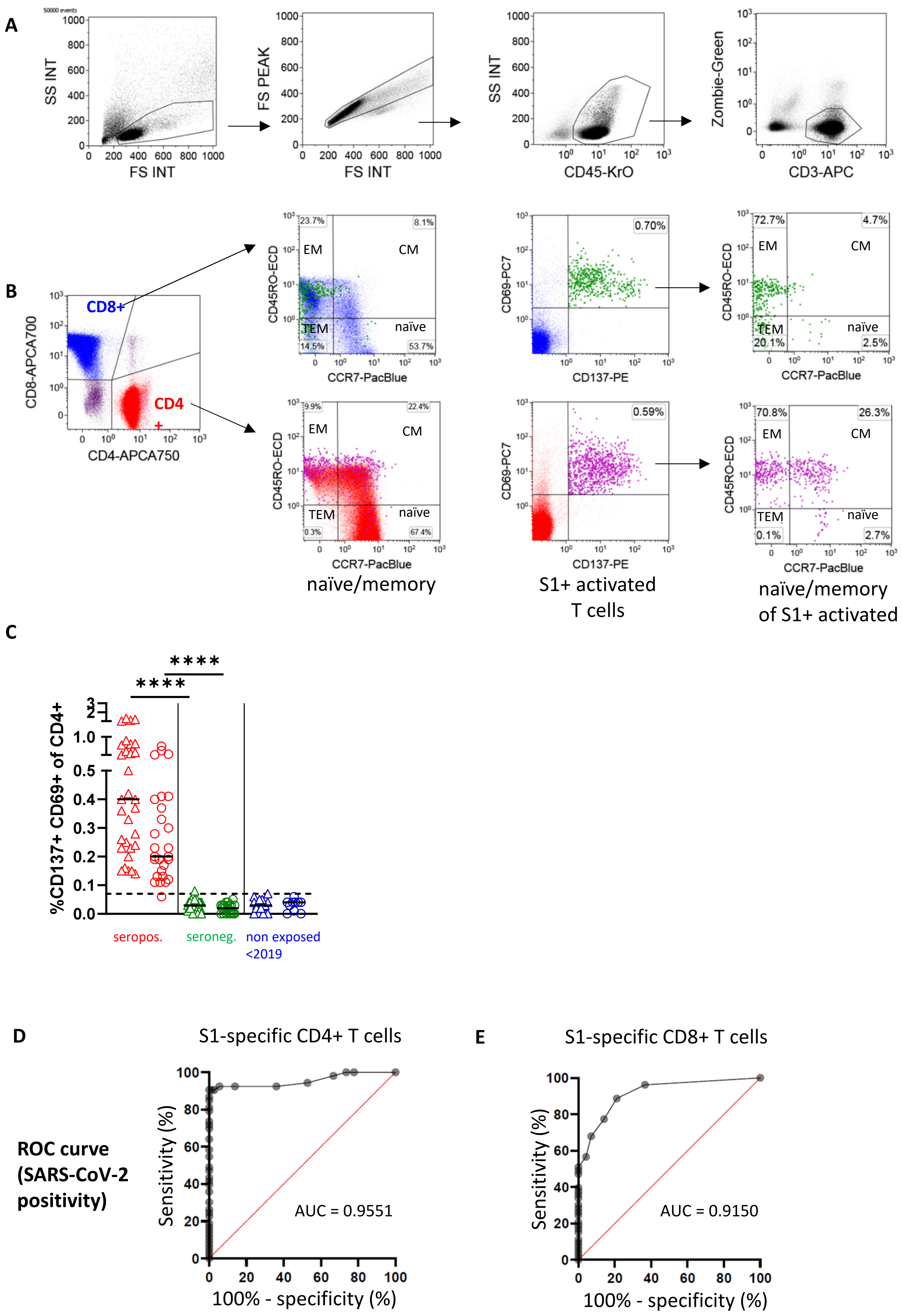

Supplementary Figure 7

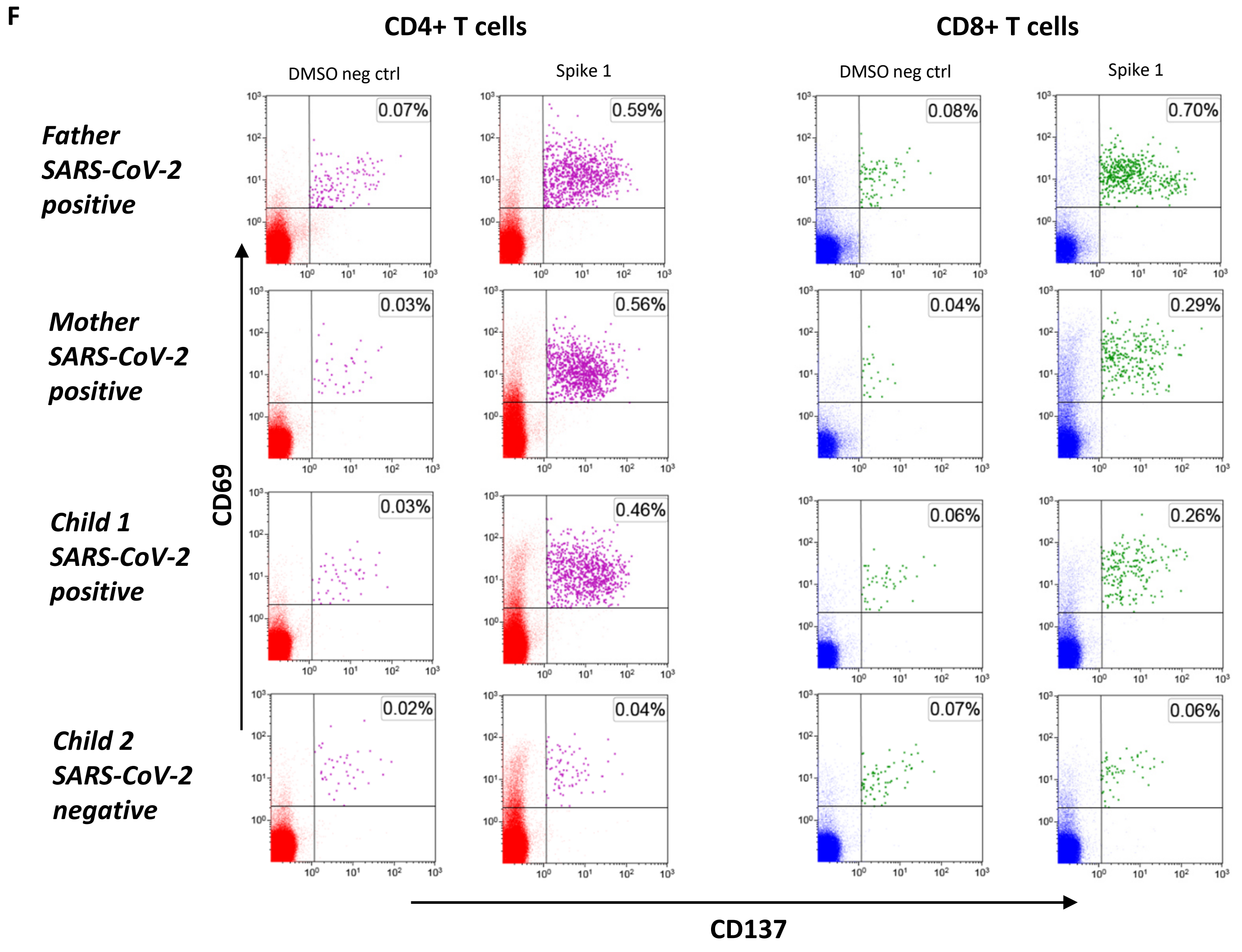

Supplementary Figure 7. Detection of S1-specific T cells and subpopulations. SARS-CoV-2 specific T cell response was analyzed after stimulation with SARS-CoV-2 Spike 1 (S1) peptide-mix for 4 days by flow cytometric detection of activation-induced markers (AIM) CD137 and CD69+. (A) Common gating strategy: the gates for CD8+ and CD4+ T cells were set after excluding doublets and gating on viable CD3+ T cells (Zombie-green negative). (B) CD8+ (upper row, blue) and CD4+ (lower row, red) T cell subpopulations were determined by using CD45RO as memory cell marker and CCR7 to distinguish central memory (CM, CCR7+) from effector memory (EM, CCR7-) and naïve (CCR7+) from terminal differentiated memory (CCR7-) T cells. S1-specific CD8+ and CD4+ T cells were determined by their co-expression of CD69 and CD137, followed by further differentiation in T-memory subpopulations by CD45RO and CCR7 staining. (C) High sensitivity and specificity of the AIM test to assess S1-specific CD4+ T cells. To show the functionality of the AIM assay, the results of three different cohort groups are shown, red: SARS-CoV-2 infected, seropositive children (circles) and adults (triangles) 4 months after infection; green: SARS-CoV2 non infected, seronegative family members; blue: non exposed adults (cells were cryopreserved before 2019). The ROC analysis (Graph Pad Prism 9.0.1) illustrate sensitivity and specificity of (D) S1-specific CD4+ T cells (sensitivity 90.6%; 95% CI: 79.8-95.9%, specificity 97.22 %; 95% CI: 90.4-99.5%) and (E) S1-specific CD8+ T cells (sensitivity 67.9%; 95% CI: 54.5-78.9%, specificity 93.0%; 95CI: 84.6-97.0%) in detection of SARS-CoV-2 infection. DMSO control values, (shown in Fig S6F) were subtracted for the calculation of specifically activated T-cells. (F) Representative dot plots of S1-specific CD4+ (red, left) and CD8+ (blue, right) of one family 4 months (T1) after infection. Mother, father and one child (#1) were seropositive for SARS-CoV-2, whereas the second child (#2) remained uninfected. Plots of CD69+CD137+ T cells after DMSO incubation for 4 days are shown as negative/ background controls. Mann-Whitney test is used for comparing median values (black lines) between infected and non infected individuals, statistical significance was defined as ****$p \leq 0.0001$. Source data are provided as a Source Data file.

Supplementary Figure 8

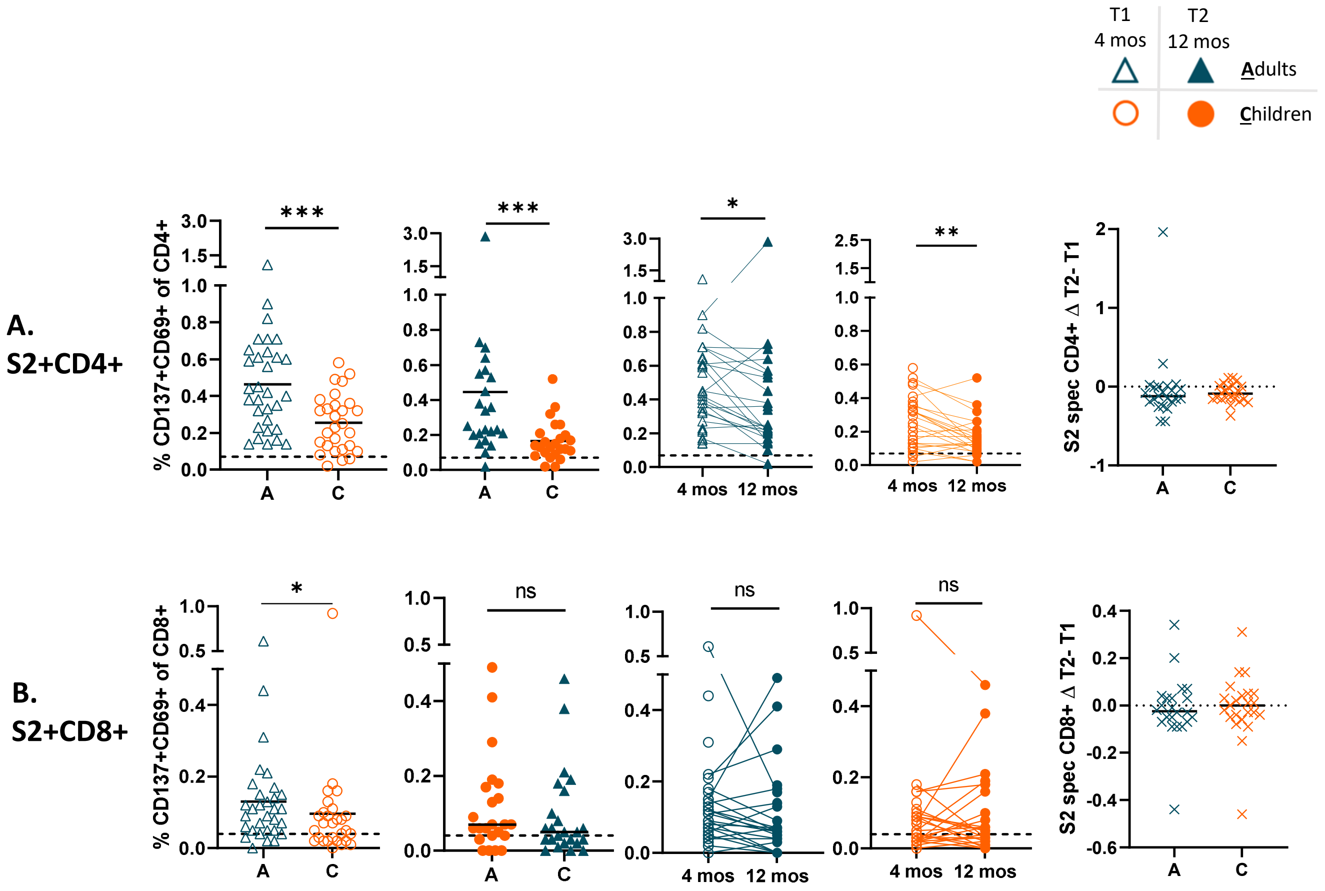

Supplementary Figure 8. **SARS-CoV-2 Spike 2 specific T-cell response is lower in children than adults but remains stable one year after SARS-CoV-2 infection in children**. The SARS-CoV-2 -specific T cell response was analyzed using an activation-induced marker (AIM) assay by flow-cytometric detection of CD69+CD137+ T cells after stimulation with SARS-CoV-2 Spike 2 peptide mix. DMSO negative control values were subtracted from AIM positive T cells after specific stimulation. The results for CD4+ (A) and CD8+ (B) of seropositive adults (blue) and children (orange) at 4 months (open symbols) and 12 months (filled symbols) after infection are shown. The dotted line in the graphs at far right depicts a null difference. Black dashed lines indicate the limit of detection of 0,07 % (CD4+) and 0,04 % (CD8+). Mann-Whitney test and Wilcoxon matched-pairs signed rank test were used for comparing median values (black lines) between adults and children, and between the two timepoints, respectively. Statistical significance was defined as * p≤0.05, **p≤0.01, ***p≤0.001, ****p<0,0001. Source data are provided as a Source Data file.

Supplementary Figure 9

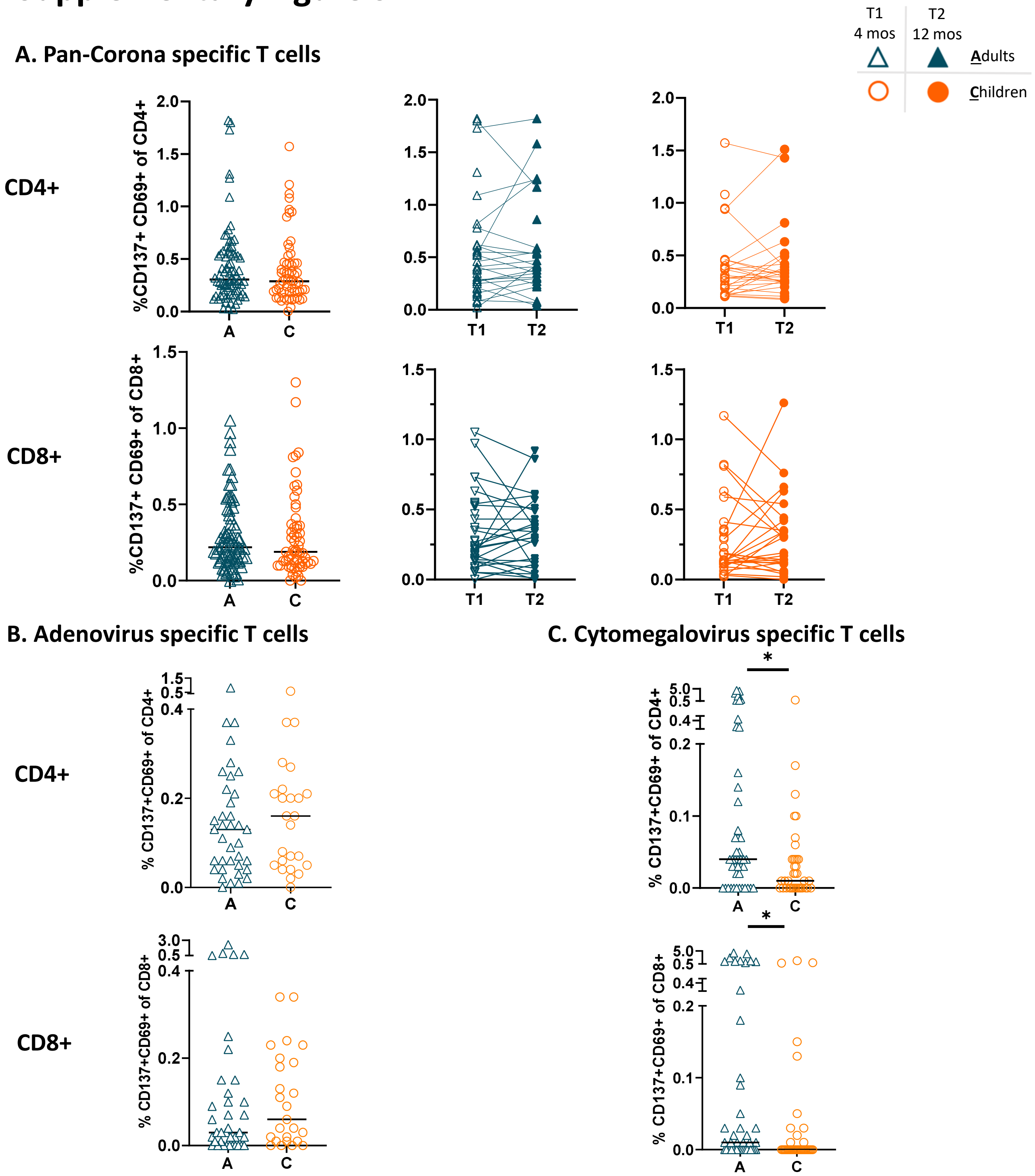

Supplementary Figure 9. Detection of virus-specific T cells after stimulation with Pan-Corona, Adenovirus (HAdV5) and Cytomegalovirus (HCMV-A). As controls for detection of SARS-CoV2 Spike 1 specific T-cells, T cells were analyzed after stimulation with pooled Spike protein peptide mixes of human common corona viruses HUK1, 229E, OC43 and NL63 ("Pan Corona" peptide mix) (A) and peptide mixes of human Adenovirus HAdV5 (B) and human Cytomegalovirus HCMVA (C). A: The amount of Pan Corona specific T cells is similar in adults and children (all family members) and doesn't change over the time after SARS-CoV-2 infection (SARS-CoV 2 seropositive family-members). B: Adults and children show similar proportions of human Adenovirus HAdV5 specific T cells. C: After human Cytomegalovirus-peptide stimulation, significantly less CMV-specific CD4 and CD8 T-cells could be detected in children. Mann-Whitney test and Wilcoxon matched-pairs signed rank test were used for comparing median values (black lines) between adults and children, and between the two time points; Statistical significance was defined as *p≤0.05, only significant results are marked. Source data are provided as a Source Data file.

Supplementary Figure 10

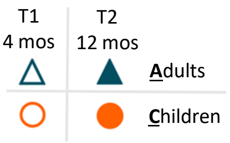

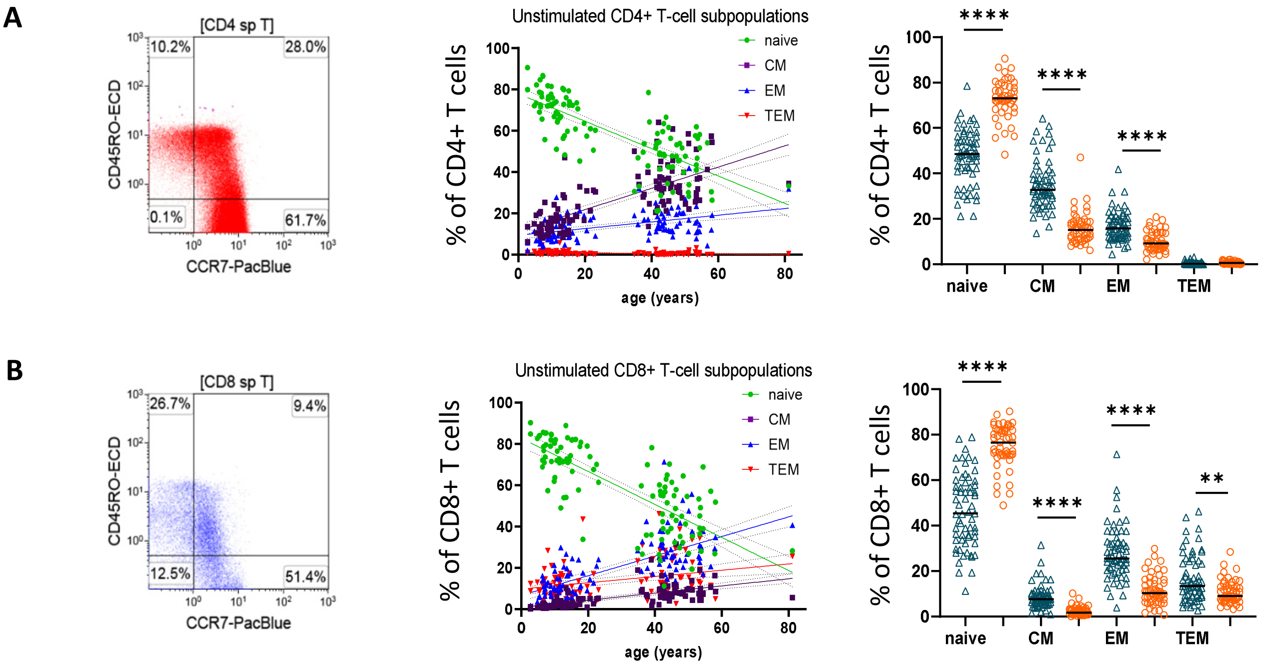

Supplementary Figure 10. Difference in distribution of the T cell subpopulations in unstimulated cells (ex vivo) between children and adults. Age-related distribution of naïve-memory T cell subpopulations of the whole cohort. Representative gating in CCR7+/CD45RO+ dot plots of CD4+ (red) and CD8+ (blue) of one individual are shown, unstimulated CD4+ (A) and CD8+ (B) According to their expression of CD45RO and CCR7, naïve T cells (CD45RO- CCR7+), were discriminated from central memory (CM) T cells (CD45RO+ CCR7+), effector memory (EM) T cells (CD45RO+CCR7-) and terminal effector memory (TEM) T cells (CD45RA- CCR7-). Mann-Whitney test was used for comparing median values (black lines) between adults and children. Statistical significance was defined as * p≤0.05, **p≤0.01, ***p≤0.001, ****p<0,0001, only significant results are marked. Source data are provided as a Source Data file.

Supplementary Figure 11

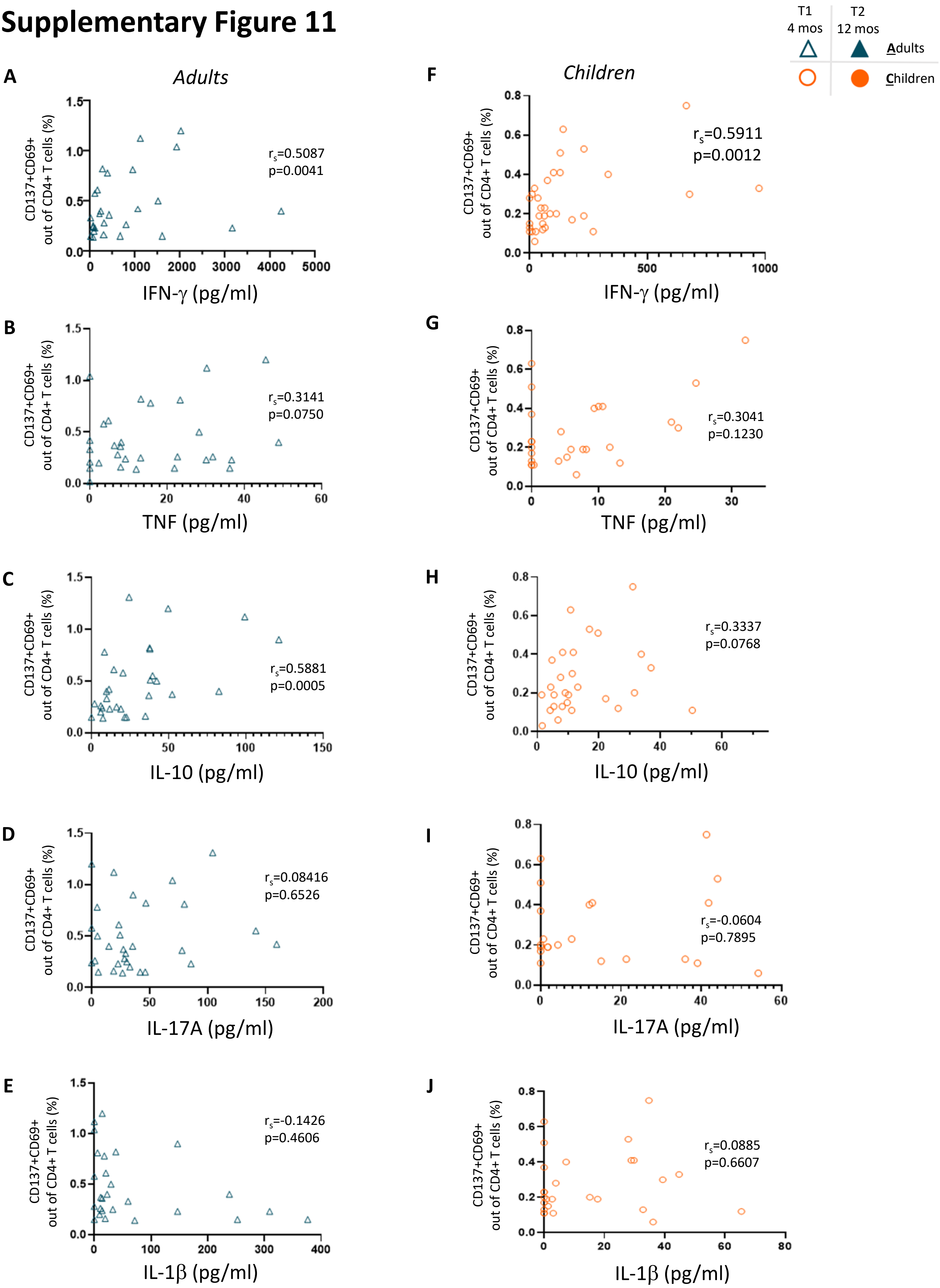

Supplementary Figure 11. The amount of the released cytokine in the supernatant of Spike-1 stimulated T cells in adults (A-E) and children (F-J) is blotted against the proportion of CD137+CD69+CD4+ T cells to analyze the correlation. While for IFN-γ (A,F), TNF (B,G) and IL-10 (C,F) there is a correlation according to the Spearman r-values (> 0,3 weak correlation, > 0,5 moderate correlation), no correlation was detected for IL-17A (D, I) and IL-1β (E,J). Source data are provided as a Source Data file.

Supplementary Figure 12

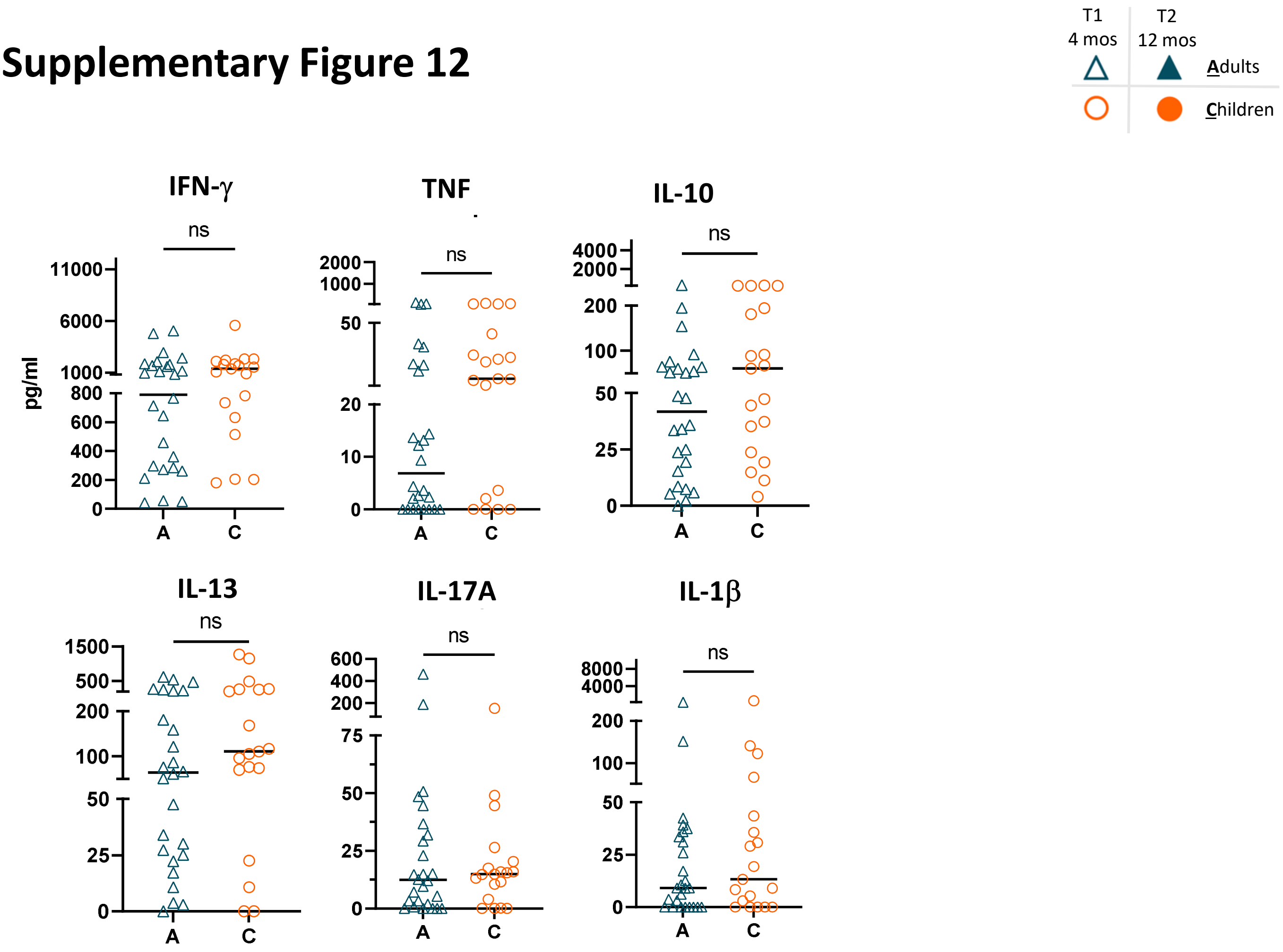

Supplementary Figure 12. Cytokine release in the supernatant of Pan Corona stimulated T cells. As a control for detection of cytokines after Spike-1-specific stimulation, the release of cytokine after Pan-Corona stimulation was analyzed in supernatants of pooled common corona viruses HUK1, 229E, OC43 and NL63 (“Pan Corona” peptide mix) stimulated T-cells by multiplex immunoassay. We found no significant differences in the release of IFN-γ, TNF, IL-10, IL-13, IL-17A and IL-1β of children in comparison to adults. Mann-Whitney test was used for comparing median values (black lines) between adults and children. ns = non-significant. Source data are provided as a Source Data file.

Supplementary Table 1

Antigen	Clone	Fluorophore	Dilution	Vendor	Lot
CD95	DX2	BV 421	1:50	BD	9080780
CD3	OKT3	SB 436	1:200	Life Technologies	1994748
CD16	3G8	SB 436	1:25	Invitrogen	2196759
CD33	WM-53	SB 436	1:50	Invitrogen	2250140
CD20	2H7	Pacific Blue	1:640	BioLegend	B316230
CD69	FN50	BV 480	1:200	BD Bioscience	156408
BAFF-R	11C1	BV 605	1:100	BD	0303183
CD38	HB-7	BV 650	1:100	BioLegend	B313181
CD27	L128	BV 786	1:80	BD	260380
IgG2	SAG2	FITC	1:40	Cytognos	2011312/2
IgG3	SAG3	FITC	1:100	Cytognos	2011316/2
IgG1	SAG1	PE	1:100	Cytognos	2011311/2
IgG2	SAG2	PE	1:80	Cytognos	2011309/2
TACI	1A1	PE-Dazzle594	1:50	BioLegend	B302174
IgA1	SAA1	PerCP-Cy5.5	1:30	Cytognos	2012570
IgA2	SAA2	PerCP-Cy5.5	1:70	Cytognos	2102067
IgD	IA6-2	PerCP-eFluor 710	1:150	Invitrogen	2198687
IgE	MHE-18	PE-Cy7	1:150	BioLegend	B306911
IgG4	SAG4	APC	1:40	Cytognos	2012541/2
IgA1	SAA1	APC	1:70	Cytognos	2012675
IgM	polyclonal	Alexa Fluor 647	1:400	Jackson ImmunoResearch	133163
CD21	Bu32	Alexa Fluor 700	1:50	BioLegend	B284903
zombie		NIR	1:800	BioLegend	B326388
CD19	HIB19	APC-Cy7	1:150	BioLegend	B311752

Antigen	Clone	Fluorophore	Dilution	Vendor	Lot
Streptavidin		BV421		Biolegend	B329652
Streptavidin		PE		Biolegend	B325169

Antigen	Clone	Fluorophore	Dilution	Vendor	Lot
CD3	UCHT1	APC	1:50	Beckman Coulter	200080, 82, 83
CD4	13B8.2	APC-Alexa Fluor 750	1:50	Beckman Coulter	200067, 69, 70, 71
CD8	B9.11	APC-Alexa Fluor 700	1:100	Beckman Coulter	200023, 24
CD45	J.33	Krome Orange	1:50	Beckman Coulter	200074, 79, 81
CD45RO	UCHL1	ECD	1:10	Beckman Coulter	200021, 22
CD69	FN50	PE-Cy7	1:20	Biolegend	B311887 B324837
CD137 (4-1BB)	4B4-1	PE	1:20	Biolegend	B311111 B323273
CD197 (CCR7)	G043H7	Pacific Blue	1:20	Biolegend	B300600
CD279 (PD1)	PD1.3	PE-Cy5,5	1:20	Beckman Coulter	200036, 37
Zombie Green		Exication 488nm	1:1000	Biolegend	B295769

Supplementary Table 1. List of all antibodies used for flow cytometry within the study.
