# Supplementary material for: High antibody levels and reduced cellular response in children up to one year after SARS-CoV-2 infection
Source: Nat Commun. 2022 Nov 28;13:7315. doi: 10.1038/s41467-022-35055-1 (PMC9701757; doi:10.1038/s41467-022-35055-1)
Supplement: Supplementary file 3 — Reporting Summary [file 41467_2022_35055_MOESM3_ESM.pdf]

nature portfolio

Corresponding author(s): Rizzi, Janda

Last updated by author(s): Oct 10, 2022

Reporting Summary

Nature Portfolio wishes to improve the reproducibility of the work that we publish. This form provides structure for consistency and transparency in reporting. For further information on Nature Portfolio policies, see our Editorial Policies and the Editorial Policy Checklist.

Statistics

For all statistical analyses, confirm that the following items are present in the figure legend, table legend, main text, or Methods section.

n/a	Confirmed	
☐	☒	The exact sample size (*n*) for each experimental group/condition, given as a discrete number and unit of measurement
☐	☒	A statement on whether measurements were taken from distinct samples or whether the same sample was measured repeatedly
☐	☒	The statistical test(s) used AND whether they are one- or two-sided<br>*Only common tests should be described solely by name; describe more complex techniques in the Methods section.*
☐	☒	A description of all covariates tested
☐	☒	A description of any assumptions or corrections, such as tests of normality and adjustment for multiple comparisons
☐	☒	A full description of the statistical parameters including central tendency (e.g. means) or other basic estimates (e.g. regression coefficient) AND variation (e.g. standard deviation) or associated estimates of uncertainty (e.g. confidence intervals)
☐	☒	For null hypothesis testing, the test statistic (e.g. *F*, *t*, *r*) with confidence intervals, effect sizes, degrees of freedom and *P* value noted<br>*Give P values as exact values whenever suitable.*
☒	☐	For Bayesian analysis, information on the choice of priors and Markov chain Monte Carlo settings
☒	☐	For hierarchical and complex designs, identification of the appropriate level for tests and full reporting of outcomes
☒	☐	Estimates of effect sizes (e.g. Cohen's *d*, Pearson's *r*), indicating how they were calculated

Our web collection on statistics for biologists contains articles on many of the points above.

Software and code

Policy information about availability of computer code

Data collection	Microsoft Excel 2016; Spectraflow (Cytek), Kaluza (Beckman-Coulter)
Data analysis	Software used: KALUZA Analysis 2.1 and FlowJo (for flow cytometry data), Graph Pad Prism 9.0 (for all statistics and graphs except heatmaps) and R version 4.1.2 using the ComplexHeatmap package (v 2.10.0). The tidyverse packages were used for data handling. The heatmap was again plotted using the ComplexHeatmap package (Wickham, H. et al. Welcome to the Tidyverse. J. Open Source Softw. 4, 1686, 2019 and Gu, Z., Eils, R. & Schlesner, M. Complex heatmaps reveal patterns and correlations in multidimensional genomic data. Bioinformatics 32, 2847–2849, 2016).

For manuscripts utilizing custom algorithms or software that are central to the research but not yet described in published literature, software must be made available to editors and reviewers. We strongly encourage code deposition in a community repository (e.g. GitHub). See the Nature Portfolio guidelines for submitting code & software for further information.

Data

Policy information about availability of data

All manuscripts must include a data availability statement. This statement should provide the following information, where applicable:

- Accession codes, unique identifiers, or web links for publicly available datasets
- A description of any restrictions on data availability
- For clinical datasets or third party data, please ensure that the statement adheres to our policy

A short version of the study protocol is available at the German Clinical Trials Register (DRKS, www.drks.de), study ID 00021521. The full study protocol is available from https://www.drks.de/drks_web/navigate.do?navigationId=trial.HTML&TRIAL_ID=DRKS00021521. Individual participant data, including data dictionaries will not be available, since we did not seek parental consent for data sharing. These data is available from the corresponding authors upon request (response within two

weeks). Requests for these data will be reviewed by the corresponding authors to verify if the request is subject to any intellectual property or confidentiality obligations. Any data and materials that can be shared will be released via a Material Transfer Agreement. Source data for each figure is provided with this paper.

Field-specific reporting

Please select the one below that is the best fit for your research. If you are not sure, read the appropriate sections before making your selection.

- [x] Life sciences
- [ ] Behavioural & social sciences
- [ ] Ecological, evolutionary & environmental sciences

For a reference copy of the document with all sections, see nature.com/documents/nr-reporting-summary-flat.pdf

Life sciences study design

All studies must disclose on these points even when the disclosure is negative.

Sample size	The sample size was based on the number of available subjects and capacity of the laboratory to performed the test. No prior calculations were done.
Data exclusions	All data from the measured subjects who were included based on inlusion criteria (s. Method section of the manuscript) was analysed and presented. No data was excluded.
Replication	The sensitivity and specificity of the methods applied were challenged with samples from seronegative (non-infected) participants. The measurements pro participant were done only once per time point. Due to limited patient material availability no replicates were done.
Randomization	N/A due to observational design of the study
Blinding	N/A due to observational design of the study

Reporting for specific materials, systems and methods

We require information from authors about some types of materials, experimental systems and methods used in many studies. Here, indicate whether each material, system or method listed is relevant to your study. If you are not sure if a list item applies to your research, read the appropriate section before selecting a response.

Materials & experimental systems

n/a	Involved in the study	
[ ]	[x]	Antibodies
[x]	[ ]	Eukaryotic cell lines
[x]	[ ]	Palaeontology and archaeology
[x]	[ ]	Animals and other organisms
[ ]	[x]	Human research participants
[ ]	[x]	Clinical data
[x]	[ ]	Dual use research of concern

Methods

n/a	Involved in the study	
[x]	[ ]	ChIP-seq
[ ]	[x]	Flow cytometry
[x]	[ ]	MRI-based neuroimaging

Antibodies

Antibodies used	Antigen/Clone/Fluorophore/Dilution/Vendor/Lot: CD95, DX2, BV 421, 1:50, BD, 9080780; CD3, OKT3, SB 436, 1:200, Life Technologies, 1994748; CD16, 3G8, SB 436, 1:25, Invitrogen, 2196759; CD33, WM-53, SB 436, 1:50, Invitrogen, 2250140; CD20, 2H7 Pacific Blue, 1:640, BioLegend, B316230; CD69, FN50, BV 480, 1:200, BD Bioscience, 156408; BAFF-R, 11C1, BV 605, 1:100, BD, 0303183; CD38, HB-7, BV 650, 1:100, BioLegend, B313181; CD27, L128, BV 786, 1:80, BD, 260380; IgG2, SAG2, FITC, 1:40, Cytognos, 2011312/2; IgG3, SAG3, FITC, 1:100, Cytognos, 2011316/2; IgG1, SAG1, PE, 1:100, Cytognos, 2011311/2; IgG2, SAG2, PE, 1:80, Cytognos, 2011309/2; TACI, 1A1, PE-Dazzle594, 1:50, BioLegend, B302174; IgA1, SAA1, PerCP-Cy5.5, 1:30, Cytognos, 2012570; IgA2, SAA2, PerCP-Cy5.5, 1:70, Cytognos, 2102067; IgD, IA6-2, PerCP-eFluor 710, 1:150, Invitrogen, 2198687; IgE, MHE-18, PE-Cy7, 1:150, BioLegend, B306911; IgG4, SAG4, APC 1:40, Cytognos, 2012541/2; IgA1, SAA1, APC, 1:70, Cytognos, 2012675; IgM, polyclonal, Alexa Fluor 647, 1:400, Jackson ImmunoResearch, 133163; CD21, Bu32, Alexa Fluor 700, 1:50, BioLegend, B284903; zombie, NIR, 1:800, BioLegend, B326388; CD19, HIB19, APC-Cy7, 1:150, BioLegend, B311752; Streptavidin, BV421, Biolegend, B329652; Streptavidin, PE, Biolegend, B325169; CD3, UCHT1, APC, 1:50, Beckman Coulter, 200080, 82, 83; CD4, 13B8.2, APC-Alexa Fluor 750, 1:50, Beckman Coulter, 200067, 69, 70, 71; CD8, B9.11, APC-Alexa Fluor 700, 1:100, Beckman Coulter, 200023, 24; CD45, J.33, Krome Orange 1:50, Beckman Coulter, 200074, 79, 81; CD45RO, UCHL1, ECD, 1:10, Beckman Coulter, 200021, 22; CD69, FN50, PE-Cy7, 1:20, Biolegend, B311887 and B324837; CD137 (4-1BB), 4B4-1, PE 1:20, Biolegend, B311111 and B323273; CD197 (CCR7), G043H7, Pacific Blue, 1:20, Biolegend, B300600; CD279 (PD1), PD1.3, PE-Cy5,5, 1:20, Beckman Coulter, 200036, 37; Zombie, Green Excitation 488nm, 1:1000, Biolegend, B295769.
Validation	Antibody were validated and titrated by test staining on PBMCs.<br>Tetramers were tested by staining samples of SARS-CoV2 infected individuals 2-4 weeks after infection. As control CD80 tetramers were used.

Human research participants

Policy information about studies involving human research participants

Population characteristics	This study examined 28 households with PCR-confirmed or seropositive symptomatic SARS-CoV-2 individuals. Only households with at least one adult or one child with history of SARS-CoV-2 infection were included. At the first timpoint (T1, approx. 4 months after SARS-CoV-2 infection) we analyzed 50 children (median age 10.4 years, IQR 7.2-13.5; 52% females) of whom 27 were positive for SARS-CoV-2. Of the 61 analyzed adults at T1 (median age 44.8 years, IQR 41.4-50.0; 47.5% females) 31 were positive for SARS-CoV-2. At the second timepoint (T2, approx. 12 after SARS-CoV-2 infection) 51 adults and 40 children from T1 were investigated. Written informed consent was obtained from adult participants and from parents or legal guardians on behalf of the children at both blood sampling time points. Participants were asked to donate a blood sample and fill in a questionnaire at both time points. Children's views on giving a blood sample were respected throughout. Blood samples and data for this sub-study were collected at the study site Ulm in July 2020 and in March 2021.
Recruitment	Participants were recruited during the first wave of the pandemic (May to July 2020) by local health authorities, public announcements and an in-hospital database of families/households with at least one confirmed PCR-positive individual. Subjects were eligible for enrolment if they met the following inclusion criteria: (i) Children (male or female) aged 0 to 18 years; (ii) parents and other adults (male or female) living in the same household with the investigated children (without age limit); (iii) residency in the state of Baden-Württemberg, Germany; (iv) written consent to the study. Key exclusion criteria were (i) severe congenital diseases (e.g. infantile cerebral palsy, severe congenital malformations); (ii) congenital or acquired immunodeficiency; (iii) no comprehension of German language. No financial compensation was provided to the participants. The data on the PCR tests for SARS-CoV-2 as well as the information on the clinical symptoms have been obtained retrospectively. The families were included only when there was at least one SARS-CoV-2-infected adult or child. This approach might cause recruitment bias.
Ethics oversight	This study was initiated by the University Children's Hospitals in Freiburg, Heidelberg, Tübingen and Ulm and approved by the independent ethics committee of each center. The study is registered at the German Clinical Trials Register (DRKS), study ID 00021521, conducted according to the Declaration of Helsinki, and designed, analyzed and reported according to STROBE guidelines.

Note that full information on the approval of the study protocol must also be provided in the manuscript.

Clinical data

Policy information about clinical studies

All manuscripts should comply with the ICMJEguidelines for publication of clinical research and a completedCONSORT checklist must be included with all submissions.

Clinical trial registration	The study is registered at the German Clinical Trials Register (DRKS), study ID 00021521, conducted according to the Declaration of Helsinki, and designed, analyzed and reported according to Strobe guidelines.
Study protocol	Study protocol (in German) is accessible at: https://www.drks.de/drks_web/navigate.do?navigationId=trial.HTML&TRIAL_ID=DRKS00021521
Data collection	Samples were collected at two separate time points, an early time point (T1) approximately 4 months post-symptom onset and a late time point (T2) approximately 12 months post-symptom onset during a planned visit at Depatment of Pediatrics of the University Medical Center Ulm.
Outcomes	Different parameters of adaptive immune response (specific antibodies in serum and saliva, specific B and T cells) at two time points were measured and analyzed.

Flow Cytometry

Plots

Confirm that:

- [x] The axis labels state the marker and fluorochrome used (e.g. CD4-FITC).
- [x] The axis scales are clearly visible. Include numbers along axes only for bottom left plot of group (a 'group' is an analysis of identical markers).
- [x] All plots are contour plots with outliers or pseudocolor plots.
- [x] A numerical value for number of cells or percentage (with statistics) is provided.

Methodology

Sample preparation	10-30 ml peripheral blood was collected in heparin tubes and peripheral blood mononuclear cells (PBMC) were isolated by density gradient separation (Pancoll separating solution, Pan-Biotech) and cryopreserved for batched analysis. Flow cytometry studies were performed on PBMCs. Antibody were added to the cell suspension, incubated for 15 minutes at 4°C.

	Then washed in PBS EDTA and resuspended in FACS buffer.
Instrument	Cytek Aurora, Cytek and Beckman-Coulter 10 color NAVIOS Flow cytometer
Software	Acquisition: Spectraflow (Cytek), Kaluza (Beckman-Coulter)<br>Analysis: FlowJo Software
Cell population abundance	No sorting was performed.
Gating strategy	For B cell phenotyping: Lymphocytes were identified by FSC-H and SSC-H gating; within this gate duplets were excluded by FSC-H-FSC-A gating; within this gate we identified live cells; within this gate lineage exclusion gate was performed gating CD19 cells against CD3/CD16/CD33; within this gate, B cells were identified with CD20 and Cd19 staining; within this gate plasmablast were identified as CD38high CD27 high; in the non plasmablast gate by CD38 and IgM we identified Transitional cells, in the non-Transitional cells by CD27 and IgD staining we identified naive, switched memory and marginal zone B cells. In the non-plasmablast cells we gated on IgD neg IgM neg and within this gate checked the subclasses.<br>Gating strategy for T cells was similar in the identification of lymphocytes single cells, live cells, then with the CD3 cells CD4 and CD8 were identified, and within those the activated ones by CD69 and CD137. Further analysis of naive and memory subpopulations was performed after gating on CD69+CD137+ activated CD4+ and CD8+T cells or unstimulated CD4+ and CD8 + T cells by using the naiv-memory discrimination markers CD45RO and CCR7.

☒ Tick this box to confirm that a figure exemplifying the gating strategy is provided in the Supplementary Information.
